# Impact of the July 2018 Heavy Rain Disaster on the Endangered Nagoya Daruma Pond Frog (*Pelophylax porosus brevipodus*) in Rice Fields of Mabi Town, Kurashiki City, Western Japan: Changes in Population Structure over Five Years

**DOI:** 10.3390/ani16030369

**Published:** 2026-01-23

**Authors:** Ryo Nakajima, Daisuke Azumi, Masakazu Tada, Junya Nakaichi, Koki R. Katsuhara, Kazuyoshi Nakata

**Affiliations:** 1 Graduate School of Environmental, Life, Natural Science and Technology, Okayama University, Tsushima-naka, Okayama 700-8530, Japanpv3y2b6g@s.okayama-u.ac.jp (J.N.);; 2 Okayama Prefectural Public Interest Incorporated Foundation for Environmental Conservation, 665-1 Uchio, Minami-ku, Okayama 701-0212, Japan

**Keywords:** agroecosystem, conservation ecology, endangered amphibian, paddy field, post-disaster habitat recovery

## Abstract

Rice paddy fields (referred to below as rice fields) are important not only for food production, but also as habitats for various species. The Nagoya Daruma Pond Frog (*Pelophylax porosus brevipodus*) is an endangered frog species endemic to Japan, mainly living in and around rice field areas. In July 2018, heavy rainfall caused severe flooding in Mabi Town of Okayama Prefecture, western Japan, submerging numerous rice fields and affecting local frog populations, including *P. porosus brevipodus*. To clarify whether the population structure of *P. porosus brevipodus* changed following the flood disaster in the rice fields of Mabi Town, we conducted quantitative field surveys in a rice fallow field in mid-October before (2017) and after (2018, 2020–2022, excluding 2019) the flood. The number of frogs declined sharply after the 2018 flood, reaching only a few individuals by 2020, but showed a substantial recovery in 2021 following the resumption of rice cultivation, although numbers decreased again in 2022. This recovery, despite fluctuations, indicates that habitat restoration through rice farming played a key role in enabling the population to rebound. Our findings underscore the importance of maintaining and restoring rice field environments after natural disasters for the survival and long-term recovery of *P. porosus brevipodus*.

## Simple Summary

The Nagoya Daruma Pond Frog is an endangered species that inhabits rice fields in western Japan. In 2018, severe flooding damaged many rice fields in Okayama Prefecture, disrupting the frog’s habitat. By comparing surveys conducted before and after the disaster, we observed changes in the number and size of frogs, along with signs of recovery once rice farming had resumed. This study highlights the importance of maintaining and restoring rice fields after natural disasters to conserve endangered species like this frog.

## 1. Introduction

Wetlands provide a variety of habitats for a diverse range of species. However, in countries and regions where urban development has progressed—such as Japan—wetlands are rapidly disappearing due to human activities [1]. Although rice paddy fields (below as rice fields) are artificial environments, they serve as alternative habitats for many wetland species [2,3]. To date, more than 5000 species have been reported to inhabit Japan’s rice fields and their surrounding areas [4]. Among these, frogs are representative species commonly found in rice fields. Many frog species spend their tadpole stage in flooded rice fields and, after metamorphosis, transition to terrestrial habitats. The diversity of organisms at lower trophic levels, such as insect prey, and at higher trophic levels, such as vertebrate predators, is considered to reflect the habitat quality for frog populations [5]. Frogs also play important ecological roles as both mesopredators and prey within terrestrial and aquatic food webs [6].

The Nagoya Daruma Pond Frog (*Pelophylax porosus brevipodus*) is a species particularly dependent on rice fields. This frog is distributed across parts of Honshu, including southern Chubu, Tokai, eastern Kinki, and eastern Sanyo regions, as well as in Shikoku, Japan [7]. This species has undergone a rapid population decline due to the conversion of rice fields to dry fields and the modification (straightening and use of concrete) of irrigation channels [8]. Thus, *P. porosus brevipodus* has been classified as an endangered species in the 2020 Red List of the Ministry of the Environment of Japan. Furthermore, because its entire life cycle is completed within rice field ecosystems, it has been designated by the Ministry of Agriculture, Forestry and Fisheries as a bioindicator species representing the biodiversity richness of rice fields [9].

Mabi Town, located in Kurashiki City, Okayama Prefecture, is a low-lying area located at the confluence of the Takahashi and Oda Rivers. Due to its geographical conditions, the area is highly susceptible to the “backwater effect,” which occurs when the water level of the Takahashi River rises due to heavy rainfall, greatly reducing the downstream flow of the Oda River and causing backflow. The History of Mabi Town [10] documents a long history of flooding and flood control measures, indicating that the area has frequently experienced flooding. In the rice fields of this region, many unimproved earth agricultural channels and levees (raised earth channel banks) remain, providing suitable habitats for *P. porosus brevipodus*.

In the habitats of Mabi Town, breeding of *P. porosus brevipodus* begins in early June when irrigation of rice field paddies starts [11]. Tadpoles can be observed in the rice fields from June until late July, during which a mid-summer drainage is typically conducted in late July. After metamorphosis, juveniles leave the water and grow while feeding in rice fields and fallow paddies. From late September to early October, the paddies enter a non-irrigated period and lose standing water, and rice harvesting occurs throughout October, with harvest dates varying among fields. Later, as temperatures drop in late October or early November, they hibernate in the soil of rice fields or fallow paddies to overwinter. Although the duration varies among individuals, overwintering generally continues until around late April of the following year [12].

From late June to early July 2018, record-breaking heavy rainfall—known as the July 2018 Heavy Rain Disaster—occurred across Japan due to the influence of the seasonal rain front and a typhoon, affecting a wide area, primarily in western Japan. During the July 2018 Heavy Rain Disaster, levee breaches occurred at two locations along the Oda River and at six locations along its tributaries, caused by rising water levels in both the Takahashi and Oda Rivers. As a result, approximately 1200 hectares of land were flooded in Mabi Town [13]. The large-scale flooding persisted from 6 July to around 8 July, and in some areas until 11 July 2018, although the duration of inundation varied among rice fields. Even after the floodwaters receded, farming activities in the affected rice fields were suspended for the remainder of the year, leaving the rice fields dried out and uncultivated.

Between July and November 2017, our previous study Tada et al. [14] conducted a capture survey of *P. porosus brevipodus* with the aim of clarifying suitable habitats of the species, prior to the July 2018 Heavy Rain Disaster. The survey was conducted at five sites within three rice fallow fields and at 18 levee locations in Mabi Town. As a result, *P. porosus brevipodus* was found to inhabit rice fallow fields and levees at high densities within the rice field zone of Mabi Town. However, these rice fields were submerged during the July Heavy Rain Disaster of July 2018, and farming activities in many of them were suspended from that time onward, resulting in significant changes to the rice paddy field environment due to drying. Habitat sites of *P. porosus brevipodus* identified in Japan have been concentrated in environments prone to geomorphological changes caused by river flooding, such as rice fields along rivers and low-lying wetlands [15]. In addition, the number of days per year with daily precipitation exceeding 200 mm has been increasing in Japan [16], thereby elevating the risk of river flooding caused by localized heavy rainfall in the habitats of this species.

Studies on the impacts of natural disasters on ecosystems, as well as the responses of animal populations to such disturbances, have been conducted in various countries, including cases of river flooding caused by heavy rainfall [17,18], debris flows in mountain streams [19], and tsunamis triggered by earthquakes [20]. These studies have provided important insights into the management and conservation of natural ecosystems following disasters. Kasada et al. [21] examined the role of biodiversity and rice paddy environments in disaster risk reduction and demonstrated the effectiveness of ecosystem-based disaster risk reduction (Eco-DRR). However, to the best of our knowledge, no studies have addressed the specific ecological issues that arise when flood disturbances impact landscape ecosystems maintained through human management (e.g., rice paddies). Flooding events, such as those that occurred in Mabi Town, may disturb habitat isolation and create conditions that promote hybridization in *P. porosus brevipodus*. Rising water levels can further facilitate dispersal and break down isolation barriers, potentially influencing hybridization dynamics [22,23]. Previous studies on species of the genus *Pelophylax* have shown that disturbances in habitat isolation can induce hybridization processes [24], which are associated with the introgression of foreign mitochondrial DNA [25]. Therefore, for the conservation of species with life histories depending on rice field ecosystems, such as frogs, it is necessary to accumulate knowledge on environmental changes in rice fields before and after flood disturbances, as well as the corresponding dynamics of animal populations.

In this study, we aimed to examine changes in the population structure of *P. porosus brevipodus* in Mabi Town before and after the July 2018 Heavy Rain Disaster by conducting quantitative capture surveys of frogs in mid-October of 2018, 2020, 2021 and 2022, at the same fallow rice field investigated by Tada et al. [14] in 2017, using the same survey effort and methodology. By tracking the population dynamics of *P. porosus brevipodus* over four years following the flood disturbance, we evaluated the impact of the natural disaster on the population structure and habitat of this frog species.

## 2. Materials and Methods

### 2.1. Study Site

**Figure 1:**
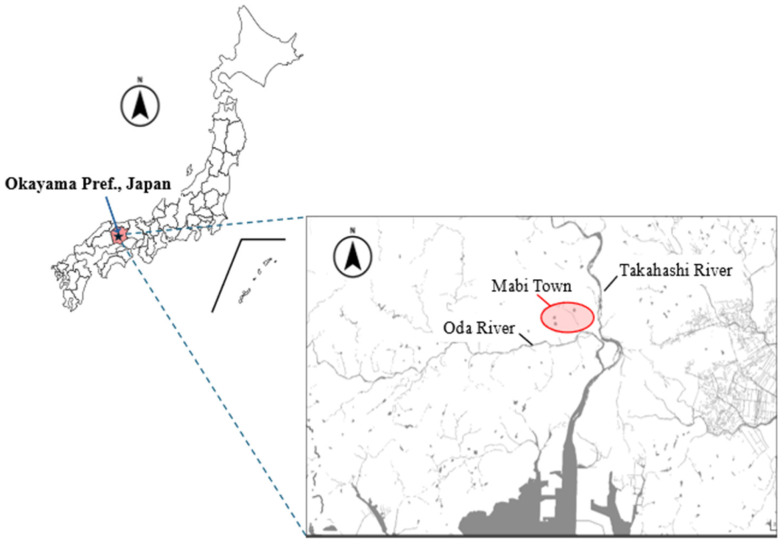
Map of the study site. The circle area indicates the habitat of *Pelophylax porosus brevipodus* in Mabi Town of Kurashiki City, Okayama Prefecture, western Japan.

We established the study area in the rice field region of Mabi Town, Kurashiki City, Okayama Prefecture, where *P. porosus brevipodus* is naturally distributed (Figure 1). In this area, many agricultural earth channels and levees have been preserved, providing suitable habitats for this species. Other frog species also inhabit this area, including the native Black-spotted Pond Frog (*Pelophylax nigromaculatus*), Indian Rice Frog (*Fejervarya kawamurai*), Wrinkled Frog (*Glandirana rugosa*), and Japanese Tree Frog (*Dryophytes japonicus*), as well as the invasive American bullfrog (*Lithobates catesbeianus*).

**Figure 2:**
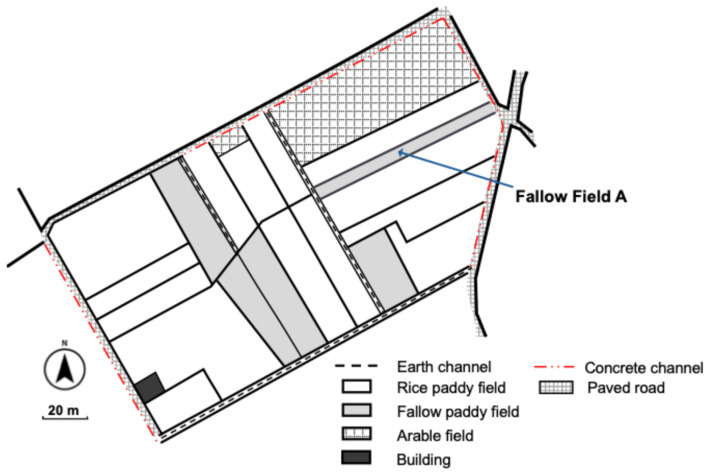
The location of the study fallow field (Fallow Field A). Part of the figure was created by modifying Figure 1 from Tada et al. [10].

**Figure 3:**
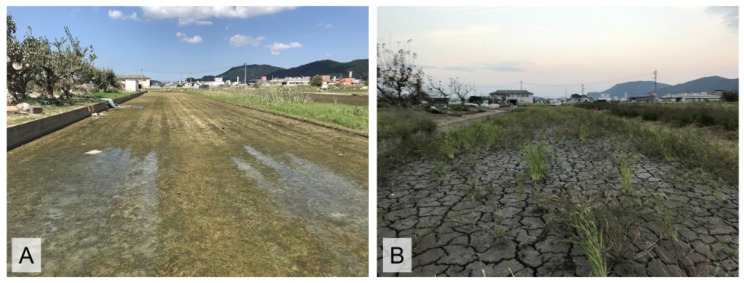
The dried-up condition of the rice paddy field adjacent to Fallow Field A after the July 2018 Heavy Rain Disaster. (**A**) 21 July 2018, about two weeks after the July 2018 Heavy Rain Disaster; (**B**) three months later, on 9 October 2018.

**Figure 4:**
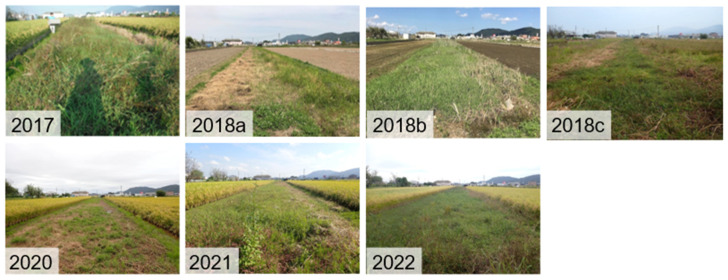
Photographs of Fallow Field A taken during surveys from 2017 to 2022, excluding 2019. All photos were taken in October except 2018a (4 June, about one month before the July 2018 Heavy Rain Disaster); 2018b was taken on 21 July (about two weeks after the disaster); and 2018c on 10 October (the regular survey date).

The survey site of this study was set in a fallow rice field (Fallow Field A in Figure 2), which had also been investigated by Tada et al. [14] for capture surveys of the species in 2017, allowing comparisons with pre-disaster data. Detailed location information of the survey site is omitted here to protect the habitat, as *P. porosus brevipodus* is an endangered species. Fallow Field A is located in the rice field zone of Mabi Town, bordered by conventionally cultivated rice fields located to the north and south, a paved road to the east, and an earth channel to the west. No structures that could impede the movement of frogs between the rice fallow field and the surrounding environment were present. Fallow Field A covers an area of approximately 570 m^2^ [10], and it is estimated that this field was inundated to a depth of about 3 m during the July 2018 Heavy Rain Disaster [26]. The surrounding rice fields to Fallow Field A were left uncultivated in 2018, during which they remained dry without water (Figure 3). However, cultivation of the rice fields surrounding Fallow Field A was fully resumed in 2019 and has been maintained continuously thereafter. Land use in the area around Fallow Field A remained unchanged before and after the heavy rain disaster. Figure 4 shows photographs of Fallow Field A taken on the survey dates from 2017 to 2022, excluding 2019 when no survey was conducted.

Fallow Field A has been left uncultivated for approximately 20 years. The land manager mows the field about two to three times a year, except in 2018 following the heavy rain disaster. In winter, dead reeds (*Phragmites australis*) dominate the area. In contrast, surrounding rice paddy fields begin irrigation between late May and early June, followed by rice cultivation. After the rice harvest in late October, these fields become no-tillage paddies covered with rice straw until the start of the next irrigation season.

### 2.2. Frog Surveys

Capture surveys of *P. porosus brevipodus* were conducted in mid-October of 2017, 2018, 2020, 2021, and 2022. Data from the 2017 survey were cited from our previous study, Tada et al. [14]. In 2017, many individuals of *P. porosus brevipodus* were observed in Fallow Field A, as well as in the surrounding rice paddy fields and levees [14]. The survey methodology followed that of Tada et al. [14], thereby enabling comparisons of the number of captured individuals and the body size composition of *P. porosus brevipodus* before and after the July 2018 Heavy Rain Disaster.

During the surveys, two researchers walked within the survey site for 30 min, capturing frog individuals using a hand net (front width: 36 cm; mesh size: 1 mm). For each captured frog, the presence or absence of vocal sacs was confirmed, and the snout-vent length (hereafter, SVL) was measured from the tip of the snout to the posterior end of the body, and recorded to the nearest millimeter. According to Matsui and Maeda [27], SVL of adult males of *P. porosus brevipodus* ranges from 35 to 62 mm (mean: 56 mm), whereas that of females ranges from 37 to 73 mm (mean: 63 mm). At metamorphosis, individuals exhibit an SVL of 22–28 mm. All frogs captured during the survey were released back into the survey site after observation.

### 2.3. Statistical Analyses

To examine differences in population structure, we fitted Gaussian mixture models to body size data and determined the number of body size groups (cohorts) for each year, excluding 2020, when the number of captured individuals was quite low. Bootstrap sampling was used to address uncertainty in the data. First, we resampled the SVL data by adding a random number from a uniform distribution between minus one and one for the number of individuals that year. Gaussian mixture models were then fitted to the resampled data, testing the number of components (k values) from 1 to 5. The Bayesian Information Criterion (BIC) was calculated for each model, and the model with the lowest BIC was selected as the best model. This bootstrap process was repeated 1000 times. Note that, because models for the 2021 data often did not converge, the same procedure was applied to that year’s data excluding the outlier (SVL = 77). Second, to determine the body size composition of each cohort, we repeated the above bootstrap procedure 1000 times for the k value that was most frequently selected as the best model for each year, and calculated the mean and standard deviation of the estimated Gaussian distributions.

All analyses were conducted in R software version 4.3.2 [28]. Model fitting of Gaussian distribution (k = 1) and Gaussian mixture models (k = 2 to 5) were implemented with the ‘fitdistr’ function in the MASS package version 7.3.60 and ‘normalmixEM’ function in the mixtools package version 2.0.0.1 of R software [28,29,30].

## 3. Results

### 3.1. Number of Frogs Captured During the Survey Period

**Figure 5:**
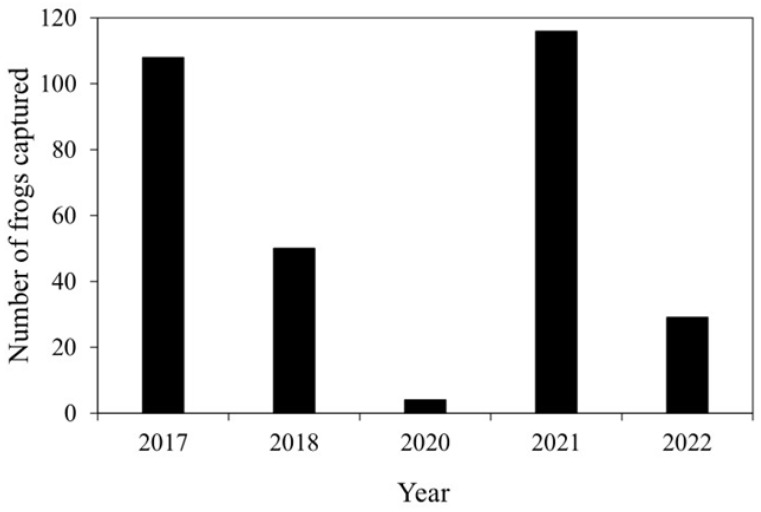
The total number of *Pelophylax porosus brevipodus* individuals captured in Fallow Field A in 2017, 2018, 2020, 2021 and 2022. The 2017 data were used, based on the dataset detailed in Tada et al. [14].

Figure 5 shows the total number of *P. porosus brevipodus* individuals captured in Fallow Field A in mid-October of 2017, 2018, 2020, 2021 and 2022. A total of 108 individuals were captured during the 2017 survey. However, in the 2018 survey conducted three months after the disaster, the number of individuals captured declined to 50, approximately half the number recorded in the previous year. Furthermore, in the 2020 survey conducted two years after the disaster, only four individuals were captured, clearly indicating that the *P. porosus brevipodus* population in Mabi Town continued to decline following the flooding. By contrast, in the 2021 survey conducted more than three years after the disaster, a total of 116 individuals were captured, representing a substantial increase compared to the previous year. In the 2022 survey, however, the number of individuals captured declined again to 29.

### 3.2. Comparison of Body Size Distribution Between the Survey Years

**Figure 6:**
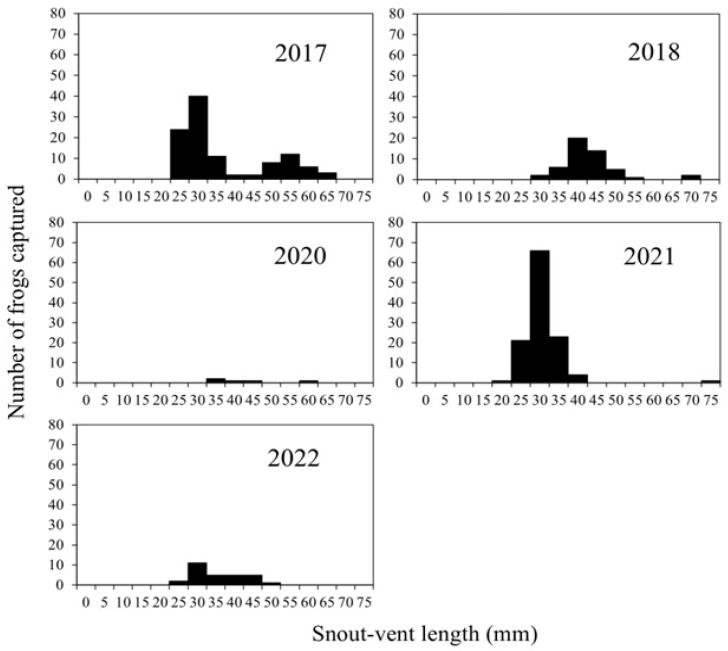
Snout-vent length (mm) of *Pelophylax porosus brevipodus* captured in Fallow Field A in 2017, 2018, 2020, 2021 and 2022. The 2017 data were used, based on the dataset detailed in Tada et al. [14].

Figure 6 shows the SVL distribution of *P. porosus brevipodus* individuals captured in Fallow Field A during the 2017, 2018, 2020, 2021 and 2022 surveys. Gaussian mixture model analysis revealed that the populations in 2017, 2018 and 2022 comprised two distinct cohorts, while the 2021 population consisted of a single cohort (Table 1). In 2017, prior to the July 2018 Heavy Rain Disaster, the mean SVLs of the two cohorts were 22.26 mm and 52.11 mm, respectively (Table 2). In 2018, three months after the disaster, the mean SVLs of the two cohorts were 39.77 mm and 52.13 mm, respectively (Table 2). In 2020, two years after the disaster, the number of individuals captured was extremely low, and model fitting was not performed. However, the captured frogs exhibited SVLs ranging from 35 to 60 mm (mean ± SE: 43.0 ± 4.6 mm). In contrast, the 2021 population, more than three years after the disaster, consisted of a single cohort with a mean SVL of 28.30 mm. In 2022, the population once again comprised two distinct cohorts, with mean SVLs of 28.52 mm and 38.89 mm, respectively.

## 4. Discussion

### 4.1. Reproductive Pattern of P. porosus brevipodus Under Pre-Flood Conditions and Breeding Response During the 2018 Heavy Rain Disaster

The breeding sites of *P. porosus brevipodus* are flooded rice fields, with breeding activity typically commencing immediately after irrigation water is introduced [31]. In the rice field zone of Mabi Town, irrigation typically begins between late May and early June, and it has been confirmed that the local population of *P. porosus brevipodus* initiates breeding soon after the irrigation begins as also found by Serizawa [11]. Breeding activity in this region continues until mid-July. Tadpoles hatched during this period undergo metamorphosis over approximately 40 days, after which they leave the rice fields and emerge onto land as juveniles.

The Heavy Rain Disaster that inundated the entire Mabi Town area occurred between 6 and 8 July 2018, coinciding with a period when most individuals of the 2018 cohort were still in the larval stage. Since amphibian larvae generally have lower environmental tolerance than adults [32], the flooding caused by the July 2018 Heavy Rain Disaster, along with subsequent changes in rice field conditions, such as the cessation of agricultural activities and the termination of the rice paddy field flooding leading to drier conditions, likely had a significant impact on the 2018 cohort of the *P. porosus brevipodus* population in Mabi Town. This interpretation is further supported by the Gaussian mixture model results, which showed that small-sized cohorts (SVL ≤ 30 mm) observed in other years were absent in 2018, indicating that tadpoles that hatched during the mid-breeding season (mid-June to early July) were largely lost to the flood.

### 4.2. Effects of the 2018 Flood, as Reflected in the Low Recruitment Observed in 2020

Our data indicates that the flood caused by the July 2018 Heavy Rain Disaster immediately washed away tadpoles and numerous adults, and that the population dynamics and age structure of *P. porosus brevipodus* became unstable after the flood. In 2017, prior to the July 2018 Heavy Rain Disaster, a large number of *P. porosus brevipodus* were confirmed to inhabit Fallow Field A [14]. However, between 2018 and 2020, the number of individuals captured declined following the disaster, indicating a reduction in the population size of *P. porosus brevipodus* in Mabi Town.

During the 2018 survey, 27 of the 50 individuals captured had SVLs of 35–45 mm. These were likely juveniles that hatched early in the breeding season (late May to early June) and completed metamorphosis prior to the July 2018 Heavy Rain Disaster. By contrast, individuals that hatched later in the breeding season were presumed to have perished or been washed away during the flooding event. By 2020, continued low capture numbers reflected the compounded effects of habitat disruption and the species’ life history, particularly the approximately two years required for females to reach sexual maturity [31], which limited the number of mature breeders and depressed recruitment.

### 4.3. Importance of the Resumption of Rice Cultivation in 2019, as Indicated by Increased Recruitment in 2021

Many rice paddy fields in Mabi Town resumed cultivation in 2019, restoring essential breeding and nursery habitats. During the 2021 survey, the number of *P. porosus brevipodus* individuals captured in Fallow Field A increased markedly compared to 2020. Among the captured individuals, 95.7% were small frogs with an SVL of approximately 30 mm. Based on the SVL data reported by Serizawa [31], these individuals were considered juveniles that had hatched in 2021. Given that most females require two overwintering periods to breed [31], these recruits were likely the offspring of females that hatched in 2019—a cohort not directly affected by the 2018 flood—indicating that the rapid restoration of rice fields enabled successful spawning in 2021.

A single female of this species is known to be able to produce approximately 1800 eggs per clutch [33]. Moreover, females have been reported to develop a new clutch of immature eggs in their ovaries following the initial spawning during the breeding season, with a subsequent spawning occurring approximately one month later [31]. *Pelophylax porosus brevipodus* is known to have a relatively long breeding season among anuran species, with breeding activity occurring from late April to mid-July [27]. However, in Mabi Town, where rice field irrigation begins in early June, the onset of the spawning season is delayed and its duration shortened; therefore, a second spawning is considered unlikely to occur [11]. Although only a small proportion of *P. porosus brevipodus* tadpoles hatched from the spawned eggs are considered to survive to adulthood, the large clutch size of this species indicates that recruitment can increase substantially within a single generation.

### 4.4. Time Lag Required for Population Dynamics to Stabilize, as Suggested by Capture Numbers up to 2022

The total number of individuals captured in the 2022 survey once again decreased to 24. This decline may be explained by the species’ life history: females of *P. porosus brevipodus* require approximately two years to reach sexual maturity [31]. Therefore, the numerous juveniles captured in 2021 were not yet reproductively mature and could not contribute to recruitment in 2022. The scarcity of mature individuals in 2022 likely reflects the sharp population decline in 2020, which resulted in a limited number of individuals reaching maturity by that year.

Nevertheless, the 2022 survey confirmed the presence of juvenile *P. porosus brevipodus*, as in the previous year, and also revealed medium-sized individuals (around 50 mm SVL), which had not been observed in the previous year. These medium-sized individuals are estimated to be one-year-old overwintered frogs [31]. From the following year onward, most of these individuals are expected to reach sexual maturity and contribute to breeding activities. These results indicate that the population, having suffered a substantial post-flood decline, exhibited early-stage recovery characterized by size- and age-structure fluctuations driven by the number of reproductively mature individuals participating in breeding; as recruitment increases under restored habitat conditions, the structure is expected to gradually stabilize.

In the study area in Mabi Town, hybrid individuals between *P. porosus brevipodus* and the Japanese brown frog (*P. nigromaculatus*) have been observed (Tada et al., unpublished data). This observation raises important questions about whether flood-induced dispersal could increase hybridization frequency and, if so, what ecological and genetic consequences might follow. Previous studies have highlighted that *Pelophylax* populations in East Asia face multiple threats, including habitat destruction, degradation of agricultural wetlands, and pollution, as well as genetic disturbances caused by hybridization [34,35]. Naito et al. [34] demonstrated that conservation efforts such as biotope creation can support the persistence of *P. porosus* populations, but also emphasized the need for long-term monitoring to prevent genetic mixing. Amin and Borzée [35] provided a comprehensive review of *Pelophylax* diversity and conservation challenges across Asia, noting that hybridization and habitat alteration are among the most critical threats to regional populations. Therefore, future research should examine whether flood-induced dispersal accelerates hybridization and assess its potential impacts on population integrity and conservation strategies.

## 5. Conclusions

Based on the findings of this study, one external factor contributing to the recovery of the *P. porosus brevipodus* population in Mabi Town by 2021, despite its marked decline in 2018, was the relatively rapid restoration of the rice field environments in the region. Many rice paddy fields resumed cultivation in 2019, the year following the disaster. If rice farming activities had not resumed in 2019, breeding sites and tadpole habitats for *P. porosus brevipodus* would have been lost for two consecutive years, and the population decline of this species would likely have been more prolonged. Therefore, for the conservation of endangered species that depend on rice field environments, it is crucial to promote rapid post-disaster restoration and to recover pre-disaster conditions as swiftly as possible in affected areas.

## Figures and Tables

**Table 1:** Results of the model selection based on BIC. The left and right sides of the slash indicate the number of models selected as the best model and of models that did not converge, respectively, with the given k value during 1000 bootstrap iterations.

	Number of Models Selected as the Best Model/Did Not Converge				
Year	k = 1	k = 2	k = 3	k = 4	k = 5
2017	0/0	788/0	171/0	33/0	8/0
2018	248/0	404/14	219/6	92/6	37/4
2021	566/0	290/314	66/310	35/294	43/270
2021 *	847/0	140/0	13/0	0/0	0/0
2022	250/0	575/0	115/0	41/0	19/5

* Data without the outlier (body size = 77) was used.

**Table 2:** Results of the Gaussian mixture model fitting. The mean value and bootstrap 95% confidence interval for the mean and standard deviation for the Gaussian distribution were calculated using 1000 bootstrap iterations.

	First Cohort		Second Cohort	
Year	Mean	SD	Mean	SD
2017	27.26 (26.51–28.12)	3.23 (2.63–3.96)	52.11 (49.30–54.28)	5.72 (3.73–8.28)
2018	39.77 (31.98–42.15)	3.89 (0.21–9.26)	52.13 (40.49–66.60)	5.29 (0.08–14.17)
2021 *	28.30 (27.69–28.92)	3.12 (2.84–3.78)		
2022	28.52 (25.88–33.17)	2.60 (0.52–6.30)	39.89 (34.71–43.23)	3.43 (0.74–6.38)

* Data without the outlier (body size = 77) was used.

## Data Availability

The data supporting the findings of this study are available from the corresponding author upon reasonable request.
